# Metabolic risk is associated with sociodemographic characteristics in adolescents from both rural and urban regions from southern Brazil

**DOI:** 10.1186/s12887-022-03386-z

**Published:** 2022-06-02

**Authors:** Sonimar de Souza, João Francisco de Castro Silveira, Kelin Cristina Marques, Anelise Reis Gaya, Silvia Isabel Rech Franke, Jane Dagmar Pollo Renner, James Philip Hobkirk, Sean Carroll, Cézane Priscila Reuter

**Affiliations:** 1 Graduate Program in Health Promotion, University of Santa Cruz do Sul, Santa Cruz do Sul, Rio Grande do Sul Brazil; 2 Graduate Program in Human Movement Sciences, Federal University of Rio Grande do Sul, Porto Alegre, Rio Grande do Sul Brazil; 3 Department of Health Sciences, Graduate Program in Health Promotion, University of Santa Cruz do Sul, Santa Cruz do Sul, Rio Grande do Sul Brazil; 4 School of Life Sciences, University of Hull, Kingston upon Hull, UK; 5 Department of Sport, Health and Exercise Science, University of Hull, Kingston upon Hull, UK

**Keywords:** Risk factors, Cardiovascular diseases, Rural health, Urban health, Metabolic syndrome

## Abstract

**Background:**

The prevalence of several cardiovascular metabolic disorders are increasingly cause for concern in adolescents worldwide. Given the complex interrelations between metabolic risk (MR) and sociodemographic variables, the present study aims to examine the association between the presence of MR with sociodemographic characteristics (sex, skin color, residential area, and parental socioeconomic status) in adolescents from Southern Brazil.

**Methods:**

Cross-sectional study conducted with 1,152 adolescents (507 males) aged between 12 and 17 years. MR was assessed using a continuous score (cMetS; sum of Z-scores of the following variables: waist circumference, systolic blood pressure (SBP), glucose, high-density lipoprotein cholesterol [HDL-C, inverse], triglycerides [TG], and estimated cardiorespiratory fitness [CRF, inverse]). Poisson regression was used to examine associations between sociodemographic variables with the dichotomized cMetS and separate metabolic variables. The results were expressed with prevalence ratios (PR) and 95% confidence intervals (CI).

**Results:**

The presence of MR (evaluated by the cMetS) was observed in 8.7% of adolescents. Higher MR was less prevalent among non-white adolescents (PR: 0.96; 95% CI: 0.93; 0.99). Adolescents living in rural areas had a lower prevalence of the following metabolic variables; low HDL-C (PR: 0.95; 95% CI: 0.94; 0.97), elevated TG (PR: 0.95; 95% CI: 0.92; 0.99), elevated glucose (PR: 0.96; 95% CI: 0.95; 0.98), and low CRF levels (PR: 0.88; 95% CI: 0.85; 0.92). Whereas, SBP was higher in those living in rural areas (PR: 1.11; 95% CI: 1.05; 1.17). In girls, there was a higher prevalence of raised TG (PR: 1.06; 95% CI: 1.02; 1.10) and lower levels of CRF (PR: 1.20; 95% CI: 1.16; 1.24), but a lower prevalence of elevated glucose (PR: 0.97; 95% CI: 0.97; 0.99).

**Conclusion:**

Higher MR prevalence was lower in those self-reporting non-white skin color and selected MR factors were less prevalent in those living in rural areas. The identification of groups at higher MR is important for early prevention and monitoring strategies for both Type 2 diabetes and later cardiovascular disease. Future studies should be conducted to assess the socio-cultural aspects of the relationships between MR and socio-cultural and lifestyle variables.

## Background

The prevalence of several cardiovascular metabolic disorders is increasingly cause for concern in adolescents worldwide. Metabolic disorders, such as hypertension, pre-diabetes, Type 2 diabetes, dyslipidemia, and obesity are continually investigated across different populations and the identification of Metabolic Risk (MR) during adolescence has played an important role in the surveillance and implications for these disorders in early adulthood [1].

MR factors and changes in metabolism, such as increased waist circumference (WC), triglycerides (TG), glucose, and total and low-density lipoprotein cholesterol (TC and LDL-C, respectively) tend to increase the presence of MR proportionally [2]. The development of these factors in childhood and adolescence, often linked to the development of insulin resistance, may represent an elevated risk of chronic cardiometabolic diseases in the longer term [3, 4]. From this perspective, identifying higher risk adolescent groups may be a relevant preventive factor for public health strategies [5]. Identification of increased MR, by characterization of the metabolic syndrome/insulin resistance syndrome, in adolescents has now been commonly recommended [6]. Nevertheless, controversy exists over which set of criteria to use among adolescents. Furthermore, evidence shows that current criteria exhibit racial/ethnic and gender differences to identify increased MR, especially within minority ethnic groups in adolescents [7].

Amongst other variables that have been investigated, levels of cardiorespiratory fitness (CRF) have been shown to contribute substantially to the MR amongst adolescents [6, 8]. Also, the presence of MR has been associated with other sociodemographic, and lifestyle aspects. Sociodemographic characteristics have been identified as strong factors for identifying MR groups. Namely, differences between the sexes, skin color, residential’ and school’ areas, type of school, and other measures of socioeconomic status have been shown to be important predictors of MR [9, 10].

Sex and residential area parameters during childhood and adolescence reflect differences in MR. Hypertension, for example, appears to be more prevalent in the rural area compared to the urban regions [11] and the excess body weight is usually higher among girls [12]. Other research findings outline the need to evaluate adolescents’ MR parameters by sex, age, and ethnic/sociodemographic groups for the prevention of health risk factors [13–15]. The prevalence of insulin resistance and associated metabolic clustering is reportedly high among Brazilian adolescents and has been associated with sociodemographic, lifestyle, dietary, anthropometric, and biochemical variables [16, 17]. Kuschnir et al. [16] reported broadly similar MS prevalence between the sexes, but higher prevalence of MS in adolescents from public schools across Brazil. This may indicate a possible association between socioeconomic factors and MS. Analysing the characteristics that may vary according to socioeconomic status, such as parenting, adiposity levels, eating patterns or sedentary time, physical activities, was suggested in the understanding of these complex relationships. Some Brazilian studies in adolescents have reported contrary findings to those found in the ERICA study, namely, a higher prevalence of MS in higher socioeconomic individuals [18], linked to higher levels of obesity and excessive screen time. Another investigation conducted within older adolescents in Santa Catarina, Brazil, reported no significant differences in age, sex, maternal education or physical activity between adolescents with and without metabolic syndrome [19].

Given these complex interrelations between MR and sociodemographic variables, the present study aims to examine the association between the presence of MR with sociodemographic characteristics in adolescents from a well-characterized sample of adolescent schoolchildren in region of Southern Brazil.

## Methods

**Fig. 1:**
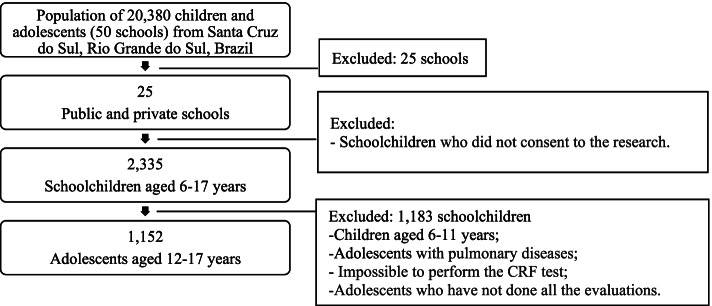
Flow chart showing the population and sampling design

The sample of this cross-sectional study came from a population of 20,380 schoolchildren. Twenty-five schools of standard educational settings (public and private network) were randomly selected from a total of 50 schools, covering the rural and urban regions of Santa Cruz do Sul, Southern Brazil. All students from the 25 schools were invited to participate in the larger study called "Schoolchildren’s Health—Phase III", developed between 2014 and 2015. Those who accepted to participate were the initial sample ranging from 6 to 17 years of age (n = 2,335; which is a representative n for the total population considering 95% confidence intervals and a margin error of 2%). Children from 6 to 11 years of age were excluded from the initial sample. Also, those who were diagnosed with respiratory disorders, unable to perform the CRF test, or were not fasting at blood collection were excluded from this sample study (Fig. 1). Thus, the present study had a final sample of 1,152 adolescents between 12 and 17 years old (645 girls), which remains as a representative n for the total population considering 95% confidence intervals and a margin error of 3%.

All evaluations were carried out in the University of Santa Cruz do Sul facilities by trained professionals. Sociodemographic characteristics, such as residential area and skin color, were evaluated using a self-reported questionnaire. The socioeconomic status was evaluated using a questionnaire fulfilled by participants’ parents. The adolescents were categorized into higher (A-B), medium (C), or low socioeconomic status (D-E) based on their parents’ household appliances (i.e.: bathrooms, automobiles, personal computers, dishwasher, etc.) and education status. Parents’ education status and each household appliance unit received a score (the more appliances, the more the score received). The final score was constructed by summing all scores, and it was categorized by the cutoff points established by the Brazilian Economic Classification Criteria [20]. The maturational stage assessment was performed using Tanner’s criteria [21], in which the adolescent self-reported the image corresponding to their current maturational status relating to pubic hair development and physical body characteristics. Five stages of maturational status for each sex were presented: stage I (pre-pubertal), stage II (initial development), stage III (continuous maturation 1), stage IV (continuous maturation 2), and stage V (matured).

The estimate of CRF was performed using the 6-min running/walking test. The distance covered by the participant (in meters) was classified into two categories: healthy zone and risk zone (high and low CRF levels, respectively), considering sex and age [22]. The WC was measured with an inelastic tape, using as reference the midpoint between the ribs and the iliac crest. Values higher than the 90^th^ percentile for age and sex were considered elevated [23]. Measurement of blood pressure was undertaken with the adolescents sitting at rest, using a manual sphygmomanometer and stethoscope, by the auscultatory method. Systolic blood pressure measures higher than the 90^th^ percentile for age, sex, and height percentiles were considered raised (borderline BP or hypertension) according to the 7^th^ Brazilian Guideline of Arterial Hypertension [24].

The blood collection (10 mL) was performed by a trained healthcare professional. Levels of 12 h fasting high-density lipoprotein cholesterol (HDL-C), TG, and glucose serum samples were analyzed on the automated Miura 200 equipment (I.S.E., Rome, Italy), using commercial Kovalent/DiaSys kits (DiaSys Diagnostic Systems, Germany). Values of TG and glucose higher or equal to 110 mg/dL and values of HDL-C lower or equal to 40 mg/dL were considered raised. These cutoff points for each metabolic component were selected using the criteria of Cook et al. [25].

MR was calculated using a continuous score (cMetS). Z-scores were computed for WC, systolic blood pressure (SBP), glucose, HDL-C, TG, and CRF, considering sex and age. The cMetS was calculated by summing the Z-scores and dividing them by six. Before cMetS calculation, HDL-C and CRF Z-scores were multiplied by -1 because of their inverse relationship with cardiovascular disease other risk factors. The presence of MR (higher MR/less favorable) was defined for values of cMetS higher than 3.40 standard deviations for boys and 3.61 standard deviations for girls, according to the previous findings of Reuter et al. [26] within the same sample of Southern Brazilian schoolchildren from the present study.

Data analysis was performed using the Statistical Package for the Social Sciences (SPSS) software, version 23.0 (IBM, Armonk, NY, USA). Descriptive data were expressed as absolute and relative frequencies. The Poisson regression was used to evaluate associations between MR and metabolic alterations with sociodemographic characteristics. The models were adjusted for maturational stage and age, and the results were expressed using prevalence ratios (PR) with 95% confidence intervals. For all analyses, values of *p* < 0.05 were considered significant.

## Results

Table 1 shows the sample characteristics. A total of 1,152 adolescents were evaluated (56.0% female and 73.0% of white skin color; 13.87 ± 1.51 years of age). The majority of the sample resided in urban areas and were categorized as middle or higher socio-economic status. The presence of higher MR was observed in 8.7% of the adolescents. Raised SBP (borderline and hypertension) was evident in 21.4% of adolescents and 14.3% exhibited elevated TG. It was notable that 68.2% of adolescents were designated in the lower CRF health risk zone for age and sex.

**Table 1:** Description of characteristics of the entire sample of adolescents (*n* = 1,152)

Age	Mean (SD)
	13.87 (1.51)
	n (%)
Sex	
Male	507 (44.0)
Female	645 (56.0)
Area of residence	
Urban	908 (78.8)
Rural	244 (21.2)
Socioeconomic level	
High (A-B)	517 (44.9)
Medium (C)	593 (51.5)
Low (D-E)	42 (3.6)
Maturational stage	
I	48 (4.2)
II	187 (16.2)
III	308 (26.7)
IV	454 (39.4)
V	155 (13.5)
Skin color	
White	841 (73.0)
Non-White	311 (27.0)
Metabolic risk	
Absence	1052 (91.3)
Presence	100 (8.7)
TG	
Normal	987 (85.7)
Elevated	165 (14.3)
HDL-C	
Normal	1115 (96.8)
Low	37 (3.2)
Glucose	
Normal	1119 (97.1)
Elevated	33 (2.9)
SBP	
Normal	906 (78.6)
Elevated	246 (21.4)
WC	
Normal	1080 (93.8)
Elevated	72 (6.3)
CRF	
Healthy zone	366 (31.8)
Increased Risk zone	786 (68.2)

Data presented using mean and standard deviation (SD) for continuous variables or absolute (n) and relative (%) frequencies for categorical variables; Maturational stage: I: prepubertal; II: initial development; III: continuous maturation; IV: continuous maturation; V: matured; *TG* triglycerides, HDL-C high-density lipoprotein cholesterol, *SBP* systolic blood pressure, *WC* waist circumference, *CRF* cardiorespiratory fitness

Table 2 demonstrates that non-white adolescents (self-reported as black, mullato, indigenous or yellow skin color) presented a lower prevalence of MR (PR: 0.96; 95% CI: 0.93; 0.99) when compared to adolescents self-reporting white skin color. The cMetS was not significantly associated with sex, residential area, or familial socioeconomic status in this sample.

**Table 2:** Association between presence of metabolic risk with sociodemographic indicators in adolescents (*n* = 1,152)

	Presence of metabolic risk	
	PR (CI 95%)	*p*
Sex		
Male	1	
Female	1.00 (0.97; 1.03)	0.839
Area of residence		
Urban	1	
Rural	0.98 (0.95; 1.02)	0.295
Socioeconomic status		
High (A-B)	1	
Medium (C)	1.03 (0.99; 1.06)	0.117
Low (D-E)	1.01 (0.94; 1.09)	0.804
Skin color		
White	1	
Non-white	0.96 (0.93; 0.99)	0.015

Poisson regression, considering as a dependent variable the metabolic risk, in a dichotomized way (absence versus presence); analyzes for sexual maturation and age. *PR* prevalence ratio, *CI* 95% confidence interval

When each MR factors was evaluated separately (Table 3), raised TG levels was more prevalent in girls (PR: 1.06; 95% CI: 1.02; 1.10). Adolescents living in rural areas had a lower prevalence of dyslipidemia, namely elevated TG (PR: 0.95; 95% CI: 0.92; 0.99) and low HDL-C (PR: 0.95; 95% CI: 0.94; 0.97).

**Table 3:** Associations between WC, TG, and HDL-C with sociodemographic indicators in adolescents (*n* = 1,152)

	Elevated WC		Elevated TG		Low HDL-C	
	PR (CI 95%)	*p*	PR (CI 95%)	*p*	PR (CI 95%)	*p*
Sex						
Male	1		1		1	
Female	1.00 (0.96; 1.03)	0.846	1.06 (1.02; 1.10)	< 0.001	0.99 (0.97; 1.01)	0.521
Residential area						
Urban	1		1		1	
Rural	1.00 (0.96; 1.05)	0.871	0.95 (0.92; 0.99)	0.024	0.95 (0.94; 0.97)	< 0.001
Socioeconmic status						
High (A-B)	1		1		1	
Medium (C)	1.00 (0.96; 1.04)	0.913	1.00 (0.96; 1.04)	0.989	1.02 (0.99; 1.03)	0.201
Low (D-E)	0.95 (0.87; 1.04)	0.279	0.98 (0.90; 1.08)	0.735	0.99 (0.95; 1.05)	0.770
Skin color						
White	1		1		1	
Non-white	0.97 (0.93; 1.02)	0.207	0.96 (0.93; 1.00)	0.077	0.99 (0.97; 1.01)	0.405

Poisson regression considering as a dependent variable WC (normal versus high), TG (normal versus high) and HDL-C (normal versus low); adjusted for sexual maturation and age. *PR* prevalence ratio, *CI* 95% confidence interval, *WC* waist circumference, *TG* triglycerides, *HDL-C* High-Density Lipoprotein cholesterol

Table 4 demonstrates that adolescent girls exhibited less prevalence of elevated glucose (PR: 0.97; 95% CI: 0.97; 0.99) and more prevalence of low levels of CRF (PR: 1.20; 95% CI: 1.16; 1.24). Adolescents living in rural areas showed a higher prevalence ​​of elevated SBP (PR: 1.11; 95% CI: 1.05; 1.17); however, lowest prevalence of elevated glucose (PR: 0.96; 95% CI: 0.95; 0.98) and CRF levels (PR: 0.88; 95% CI: 0.95; 0.92).

**Table 4:** Association between glucose, SBP, and CRF with sociodemographic indicators in adolescents (*n* = 1,152)

	Elevated Glucose		Elevated SBP		Low CRF	
	PR (CI 95%)	*p*	PR (CI 95%)	*p*	PR (CI 95%)	*p*
Sex						
Male	1		1		1	
Female	0.97 (0.97; 0.99)	0.012	0.99 (0.95; 1.03)	0.703	1.20 (1.16; 1.24)	< 0.001
Residential area						
Urban	1		1		1	
Rural	0.96 (0.95; 0.98)	< 0.001	1.11 (1.05; 1.17)	< 0.001	0.88 (0.85; 0.92)	< 0.001
Socioeconomic status						
High (A-B)	1		1		1	
Medium (C)	1.00 (0.98; 1.02)	0.713	1.03 (0.99; 1.07)	0.166	0.99 (0.96; 1.02)	0.650
Low (D-E)	1.00 (0.96; 1.05)	0.863	0.98 (0.89; 1.08)	0.714	0.95 (0.88; 1.04)	0.265
Skin color						
White	1		1		1	
Nonwhite	0.98 (0.97; 1.00)	0.124	1.02 (0.98; 1.07)	0.284	1.01 (0.97; 1.04)	0.633

Poisson regression considering as dependent variable glucose (normal versus high), SBP (normal versus high), and CRF (normal versus low); regression analyses adjusted for sexual maturation and age. *PR* prevalence ratio, *CI* 95% confidence interval, *SBP* systolic blood pressure, *CRF* cardiorespiratory fitness

## Discussion

This study indicated that MR was associated with skin color within adolescents from a well-defined geographical area of southern Brazil. When MR components were analyzed individually, adolescents living in rural areas showed a lower prevalence of raised TG, glucose, and CRF, and low HDL-C. However, they did exhibit a higher prevalence of elevated SBP. Concerning the association of risk factors by sex, female adolescents had a similar prevalence of overall MR to male adolescents, despite presenting a higher prevalence of raised TG and low CRF.

The presence of increased cumulative MR in the study was demonstrated in 8.7% of the sample of adolescents. Within an earlier published study from this sample, the prevalence of metabolic syndrome (using pre-defined MR factors criteria), was only 1.9% in adolescents [5]. The large cross-sectional, multi-ethnic Brazilian ERICA (Study of Cardiovascular Risk in Adolescents) evaluated the prevalence of MR factors that constitute the metabolic syndrome in a representative sample of over 37,500 adolescents (60.0% female) aged from 12 to 17 years. Using the non-adult International Diabetes Federation (IDF) definition for the metabolic syndrome the prevalence was typically slightly higher (2–3%) across sex and adolescent age-groups. The prevalence of MS in the South region of Brazil was higher than in the other regions, mostly due to the prevalence observed in the cities with more than 100,000 inhabitants in this macro-region [16].

Also, MR in our study appears to be higher in those adolescents self-reporting a white skin color. Similarly, Caucasian North-Americans demonstrated a stronger association with MR [27] than other racial/ethnic groups. Using 1999–2010 data from the National Health and Nutrition Examination Survey (NHANES), Gurka et al. [28] performed a confirmatory factor analysis of a single metabolic syndrome factor amongst 4,174 male and female non-Hispanic blacks, non-Hispanic whites, and Hispanics aged 12–19 years old. This evaluation allowed differential loadings across sex and race/ethnicity, resulting in a cMetS. Loadings to the score differed by racial/ethnic and gender subgroup, mainly with respect to the dyslipidemia component of the syndrome, namely TG and HDL-C.

Among 7,385 adolescents aged 12 to 17 years from the United States assessed within the National Health and Nutrition Examination Survey (NHANES), a close relation of MR with the anthropometric measurement of abdominal obesity, namely the waist-height ratio was observed [29]. A cross-sectional study conducted with 1,069 Brazilian adolescent participants (the Cardiovascular Risk in Adolescents Study) aged 12–17 years, showed that all commonly applied anthropometric indices, especially the waist-to height ratio, had excellent predictive capacity for metabolic syndrome characteristics [30]. Also, insulin resistance (determined using the 75^th^ percentile of the Homeostatic Model Assessment for Insulin Resistance; HOMA-IR) was detected in 27% of the adolescents and was more prevalent among younger adolescents (12 to 14 years), those residing in the southern and south-eastern regions of the country, and those who were physically inactive [17]. Regarding the lipid profile, de Andrade et al. [17] found substantial proportions of individuals with borderline and high levels of TG, in addition to low levels of HDL-C. Cunha et al. [31] analysed the lipid profile of 600 adolescents (10 to 19 years of age) in the state of Paraná (south Brazil) and found altered lipid levels, and high proportions of low HDL-C (52%) and borderline or high TG (30%), relatively consistent with the frequencies reported in the ERICA investigation (46.6 and 19.8%, respectively). In the adjusted analysis of the lipid variables, only TG was associated with insulin resistance. It seems that TG levels increase with the advancement of puberty in both sexes. As expected, the prevalence of insulin resistance was 2.5-fold higher among individuals categorised with severe obesity, with both higher waist circumference and high serum TG levels associated with a higher likelihood of insulin resistance.

Gonçalves et al. [32] showed significant contextual and individual characteristics related to the school food environment were associated with hypertension and obesity in Brazilian adolescents. The importance of the offer and consumption of planned meals and the negative influence of the purchase of poorer quality foods at school cafeterias were identified in relation to these MR factors.

An in-depth discussion of the role of skin color in the prevalence of higher blood pressure was carried out by Gravlee and Dressler [33]. Interestingly, in an analysis carried out by reflectance spectrophotometry, no relationship was observed between blood pressure levels and skin pigmentation. These data highlight the importance of the differences between the individual's perception of their skin color and the real pigmentation of the skin and also shows the mediating role of sociocultural factors since individuals with dark self-ratings of color who have lowered or high socioeconomic status have different blood pressure levels. These hypotheses underscore the importance of the interaction of socio-cultural processes in the relationship between skin color and blood pressure disorders.

Adolescents resident in rural areas showed a lower prevalence of raised TG, HDL-C, glucose, and low CRF, and a higher prevalence of high SBP in our study. Living habits in urban and rural regions differ mainly by the environment by allowing different forms of food, physical activity, and access to relevant healthcare and information/advice. In a separate analysis of adolescents in the Brazilian ERICA study [34], multilevel logistic regression models of individual characteristic of the adolescent and school environment were undertaken in relation to the coexistence of behavioral risk factors for cardiovascular diseases. School-level variables show that studying in private schools and living in economically favoured Brazilian regions have increased adolescents’ likelihood of belonging to higher cardiovascular disease behavioural risk profile. The coexistence of behavioural patterns encompassed risk factors such as smoking, alcohol intake and levels of highly processed food/drink intake. Based on the analysis applied to school environment variables, it was possible seeing increased likelihood of coexistence of risk factors for cardiovascular disease in adolescents studying in private schools located in socioeconomic developed Brazilian regions.

In a Venezuelan study, the altered TG/HDL-C ratio in children and adolescents may be a prediction for MR [35]. In Iran, low HDL-C prevalence in children and adolescents was higher in urban than in rural areas, demonstrating that there were differences between the residential areas [36]. Hypertension was present in adolescents in rural areas in northeastern China when compared with the urban area. Differently from a Chinese multicenter study, in which the mean SBP was higher in the urban area [11]. Data from a study conducted in Taiwan, with 649,442 adolescents, is consistent with our findings, in which lower values ​​of CRF were presented in those rural adolescents [37]. On the other hand, in a study with Chinese children and adolescents, there was a stronger association of raised glucose in those residing in the urban zone. It may be inferred that mainly lifestyle factors, including physical activity status and dietary habits, can lead to changes in these social indicators of metabolic health.

Further, this study demonstrated that high glucose was less prevalent and that elevated TG was more prevalent in female adolescents. In contrast, Agostinis-Sobrinho et al. [38], in a study conducted in the north of Portugal, demonstrated that girls had lower TG values ​​compared to boys. In the United States, a retrospective study of 8,337 adolescents in North Carolina showed that girls tend to have a lower risk of increased glucose or pre-diabetes [39]. In Europe, a lower prevalence of pre-diabetes in girls was found, similar to our findings [40]. Also, the National Health and Nutrition Survey 2005–2014 showed that boys have a higher prevalence of increased glucose and pre-diabetes (21.4%) [41].

Our study indicated that girls have a higher prevalence of low CRF levels compared to boys. In Japan [42] and Portugal [38], lower levels ​​of CRF have also been identified in girls. In a Brazilian study, low levels of CRF were associated with MR [43]. More recently, both moderate-to-vigorous physical activity and sedentary time have been shown to be independently associated with MR in Brazilian adolescents [44]. These changes are a reflection of lifestyle, unhealthy environmental, and social factors that can lead to negative results of metabolic health [45]. It is suggested that the early identification of MR in adolescence is fundamental to the prevention of cardiovascular diseases later during adult life.

This form of assessment using cMetS allows us to effectively cluster more than one MR factor into a single indicator, demonstrating the presence of MR in adolescents in the different sociodemographic groups. However, as a limitation of this study, glucose assessment is considered an indirect method of measuring insulin resistance—seen by many investigators as an important mediator of MR during adolescent growth and development. Second, the cross-sectional design did not make it possible to follow the evolution of health conditions prospectively. Also, it was not possible to include a comprehensive assessment by ethnic groups (assessed by skin color self-evaluation), as the adolescents were predominantly white. Lastly, adolescents should make more than one blood pressure assessment before classification, since a single assessment may overestimate the blood pressure values and prevalence [46].

## Conclusion

The MR presence was associated with skin color. Also, some relevant differences between the residential area, sex, and skin color were identified for selected MR variables: residential area was associated TG, HDL-C, glucose, SBP, and CRF, whereas sex was associated with TG, glucose, and CRF. The presence of MR factors in adolescents indicates an unavoidable and worrying risk for future cardiovascular complications. Moreover, the presence of cumulative MR in adolescents was comparatively high. These findings demonstrate the need to prevent metabolic deterioration at this early age. Thus, this study encourages the prevention of MR in adolescents and the monitoring of MR by public health agencies. Future studies should evaluate longitudinal MR factors in different populations, considering other ethnic groups and sociocultural factors.

## Data Availability

The database used and analyzed in the present study is not publicly available as its information may compromise the participants’ privacy and consent involved in the research. However, the data are available from the corresponding author (EA), upon reasonable request.

## Abbreviations

cMetS: Continuous metabolic risk score
CRF: Cardiorespiratory fitness
HDL-C: High-density lipoprotein cholesterol
LDL-C: Low-density lipoprotein cholesterol
MR: Metabolic risk
SBP: Systolic blood pressure
TC: Total cholesterol
TG: Triglycerides
WC: Waist circumference
